# Influence of Correspondence Noise and Spatial Scaling on the Upper Limit for Spatial Displacement in Fully-Coherent Random-Dot Kinematogram Stimuli

**DOI:** 10.1371/journal.pone.0042995

**Published:** 2012-10-09

**Authors:** Srimant P. Tripathy, Syed N. Shafiullah, Michael J. Cox

**Affiliations:** School of Optometry and Vision Sciences, University of Bradford, Bradford, West Yorkshire, United Kingdom; University of Melbourne, Australia

## Abstract

Correspondence noise is a major factor limiting direction discrimination performance in random-dot kinematograms [1]. In the current study we investigated the influence of correspondence noise on Dmax, which is the upper limit for the spatial displacement of the dots for which coherent motion is still perceived. Human direction discrimination performance was measured, using 2-frame kinematograms having leftward/rightward motion, over a 200-fold range of dot-densities and a four-fold range of dot displacements. From this data Dmax was estimated for the different dot densities tested. A model was proposed to evaluate the correspondence noise in the stimulus. This model summed the outputs of a set of elementary Reichardt-type local detectors that had receptive fields tiling the stimulus and were tuned to the two directions of motion in the stimulus. A key assumption of the model was that the local detectors would have the radius of their catchment areas scaled with the displacement that they were tuned to detect; the scaling factor *k* linking the radius to the displacement was the only free parameter in the model and a *single* value of *k* was used to fit all of the psychophysical data collected. This minimal, correspondence-noise based model was able to account for 91% of the variability in the human performance across all of the conditions tested. The results highlight the importance of correspondence noise in constraining the largest displacement that can be detected.

## Introduction

A random-dot kinematogram (RDK) consists of two, or more, frames of randomly positioned dots, with a proportion of the dots not being randomly positioned but being displaced coherently between any two temporally adjacent frames by an amount selected from a defined and limited range of distances and directions (e.g. [2]–[6]). The proportion of dots that are coherently displaced is referred to as the *coherence level*. The primate visual system is extremely sensitive to motion in RDKs and under optimal conditions, coherence levels at the threshold for discriminating direction of motion (*coherence thresholds*) can be lower than 5%. RDKs have been a very useful tool for understanding motion processing in human vision because they help us to disambiguate the use of motion cues from position cues [7], [8]. They have also been useful for understanding neural mechanisms underlying the motion processing system in primates, notably in cortical area MT/V5, and for investigating the links between physiology and psychophysics of motion perception (e.g. [9]–[18]; see also [19]–[22]).

A variety of measures have been used to evaluate human performance for detecting motion with RDK stimuli. These performance measures include:

- *coherence threshold* – the smallest proportion of dots that must be moved in a fixed direction for coherent motion to be reliably perceived (e.g. [1], [23]);
- *Dmax* – the largest displacement of the coherently moved dots for which motion is reliably perceived (e.g. [2], [5], [24]–[26]);
- *Dmin* – the smallest displacement of the coherently moved dots for which motion is reliably perceived (e.g. [7], [27], [28]); and
- *Tmax* – the largest temporal interval between successive frames for which motion is reliably perceived (e.g. [25], [29]).

Understanding how each of these measures is influenced by changes in stimulus parameters and the links between these different measures is important for integrating our understanding of the mechanisms underlying human motion detection.

In order to successfully identify the direction of motion from adjacent frames of an RDK, the visual system must solve the *correspondence problem*, i.e. it must determine the dot-displacement between frames that maximises the overlap between dots in adjacent frames ([1], [30]–[36], see also [37]–[39]). The correspondence problem exists because any dot in one frame can be paired with any dot in the adjacent frame, but only a small proportion of the potential pairs are signal pairs. The remaining pairings are spurious pairs, some of which can potentially be confused with the signal pairs, yielding *correspondence noise* (see [1]). Barlow and Tripathy [1] measured coherence thresholds for a wide range of stimulus parameters and showed that correspondence noise is a major factor influencing threshold coherence in RDKs. The issue of correspondence noise in RDK stimuli has also been studied with the Dmax paradigm (e.g. [36], [40], [41]). The purpose of this paper is to further investigate the role of correspondence noise in motion perception for RDK stimuli, particularly with regard to its influence on Dmax.

According to the correspondence noise explanation, Dmax is the maximum pattern displacement that can be detected before mispairings of dots across frames causes coherent direction discrimination to be compromised. The role of correspondence noise in motion perception in RDKs has been investigated by manipulating the probability of false-matches by varying dot-density [5], [40], [42], [43] or by manipulating some spatial- or spatiotemporal-feature (such as polarity of some of the dots, dot lifetime in multiple frame stimuli, etc.) of the dots between frames (e.g. [36], [44]). The general finding of these studies is that manipulations that reduce the probability of dot-mismatch across frames result in increases in Dmax. Dmax increases linearly with 2D element spacing [40]. It is now accepted that the information limit/correspondence noise is the critical variable limiting Dmax.

A constraint in previous attempts to model Dmax was that the coherence level used in the stimuli modelled was fixed at 100%, i.e. all the stimulus dots were moved coherently in one of two directions (a few exceptions are – [45], [46]). The goal of the current study is to present an approach that investigates Dmax at a coherence level of 100% but could be easily generalised to investigate Dmax at low coherence levels, such as 30%. The current paper presents psychophysical data and simulation results for Dmax for RDK stimuli with 100% coherence. Results for RDK stimuli with 30% coherence are presented in [47].

Barlow and Tripathy [1] investigated the influence of correspondence noise on coherence threshold. In that study psychophysical performance was reported for the displacement-size that yielded optimal performance and the influence of correspondence noise for other displacement-sizes was not systematically investigated. The current study presents psychophysical data for identifying the direction of RDK-motion for a wide range of dot densities and displacement-sizes and presents a minimalistic Reichardt-type correlator model for Dmax which readily generalises to coherence levels of less than 100%. Simulations using this model are presented for RDK stimuli with 100% of the dots being coherently displaced. Our model, which effectively uses just one free parameter, is able to simulate psychophysical performance for detecting motion in RDKs over a wide range of stimulus parameters.

## Methods

### A. Psychophysics

#### Equipment

RDK stimuli were generated on an Apple G3 Macintosh computer (Apple Computer International, Cork, Ireland) and displayed on a Formac, Pronitron CRT monitor 21/650 with a refresh rate of 70 Hz. Screen resolution was 1600 (H) × 1200 (V), with each pixel subtending 1 × 1 arcmin in the horizontal and vertical directions at a viewing distance of 82 cm. Chin and forehead rests were used to constrain head movements and to ensure a constant viewing distance. Motion in the RDKs was either leftward or rightward; observers used the left- and right-arrow keys to report the direction of perceived motion. Computer programs, written in C language running within the Vision Shell software environment [48], displayed the stimuli and collated the responses. The experiment was conducted in a very dimly-lit room, illuminated by an angle-poise lamp pointing away from the observer and facing a black screen. The dim lighting minimised screen reflections while providing sufficient ambient light to prevent fluctuations in accommodation.

#### Stimulus

Throughout the study only 2-field RDKs were used; having a larger number of fields would have introduced correspondence between temporally non-adjacent as well as adjacent fields, and the efficiency for detecting motion would have fallen [1]. Each field of dots was presented for 157 ms (11 video frames). No frames separated the two fields (interstimulus interval (ISI) was 0 ms), as performance for identifying motion direction has been shown to be best at this ISI, regardless of dot-density, when the dot-displacement is smaller than 50 arcmin ([42], see also [36]). Each dot in the two fields was a square of 4×4 arcmin, with a luminance of 76.8 cd/m^2^ against a background luminance of 0.1 cd/m^2^. The frame of the monitor was visible in the ambient illumination and acted as a peripheral fixation target.

In each trial, the motion contained in the RDKs was in the horizontal direction, randomly selected to be right or left with equal probability. Following presentation of the two fields the screen returned to the background luminance until the observer responded. After the observer’s response, feedback was presented in the form of a high/low audio tone for a correct/incorrect response, followed by a delay of 1500 ms before the next trial was started. The long inter-trial delay minimised, as far as possible, the chances that motion in one trial interfered with that in the next and minimised accumulation of motion adaptation from repeated trials. In addition, the random interleaving of the two directions of motion would have reduced the accumulation of motion adaptation and after-effects [49]. However, motion after-effects are not certain to have been eliminated entirely because these have been shown to occur even for adaptation durations as brief as 25 ms [50].

Each field of dots was presented over a stimulus area of 1600 (W) × 600 (H) pixels, centred on the screen. In the first field, the dots assigned for coherent displacement were confined to the central 900 pixels, horizontally. Therefore, the effective stimulus area (ESA) was 900 × 600 pixels (i.e., 900 × 600 arcmin when the viewing distance was 82 cm). In the experiments described in the current paper coherence level was 100%, i.e., all the first field dots lying within the ESA were coherently displaced a fixed distance right/left into the second field. The stimulus area outside the ESA in the two fields [the two flanking regions to the right and left of the ESA, each (350±displacement size) pixels wide] had randomly positioned noise dots of the same dot-density as within the ESA.

In the absence of the noise dots flanking the ESA the movement of the two vertical edges of the ESA would indicate the direction of motion of the dots. The function of the noise dots was to mask the movement of the ESA-edges. The traditional approach to prevent the movement of the stimulus edge is to “wrap-around” the dots moving out of a fixed stimulus area (e.g. [5]). However, this wrap-around procedure has a few limitations; the wrapped-around dots reduce the number of signal dots available and produce motion signals in the direction opposite to the coherent motion in the stimulus. The procedure of using flanking noise dots on either side of the ESA ensured that the number of signal dot-pairs within the ESA could be exactly controlled, regardless of the size of the displacement. It however has the disadvantage that the “motion-edge” separating coherent motion within the ESA and non-coherent noise outside the ESA was sometimes discernible when viewed directly. To ensure that the “motion-edge” could not be used to discriminate the direction of motion the stimulus was elongated along the axis of motion (see also [51], [52]), pushing the “motion-edge” further into the periphery.

#### Procedure

The stimulus on each trial consisted of a 2-field RDK with rightward or leftward motion. The observer viewed the stimulus monocularly and reported the perceived direction of motion, right or left, using the arrow keys on the keyboard. Audio feedback was provided following each trial and the next trial was initiated. All stimulus parameters, apart from the direction of motion, were fixed within a block; between blocks we varied dot-displacement and dot-density. Each block of trials consisted of 10 practice trials and 45 recorded trials. The practice trials were not used in the analysis; their main function was to help observers adjust to the randomised changes in stimulus conditions between blocks.

Dot-density was varied between blocks by varying the number of dots within the ESA, which was of fixed area. The number of dots within the ESA was selected from: 20, 40, 80, 160, 320, 640, 1280, 2560 and 4000. The displacement sizes were: 50, 60, 80, 100, 125, 150, and 200 arcmin. For each dot-density and dot-displacement combination, three blocks were run, yielding 135 recorded trials per condition. A displacement size was randomly selected, and 27 blocks (three cycles of the nine randomly-ordered densities) of data were collected with this displacement. This was repeated at each of the 7 dot displacements in random order. The performance measure, for each combination of dot-density and dot-displacement, was the proportion of trials for which the direction of motion was correctly identified.

#### Observers

The three authors participated as observers. MC and ST had normal vision, while observer SN was mildly amblyopic (VA = 6/12) in his left eye. All data collected was under monocular conditions, each observer using his dominant eye. All observers were fully aware of the experimental procedures involved and willingly consented to their participation in the study.

### B. Modelling

#### Model description

**Figure 1:**
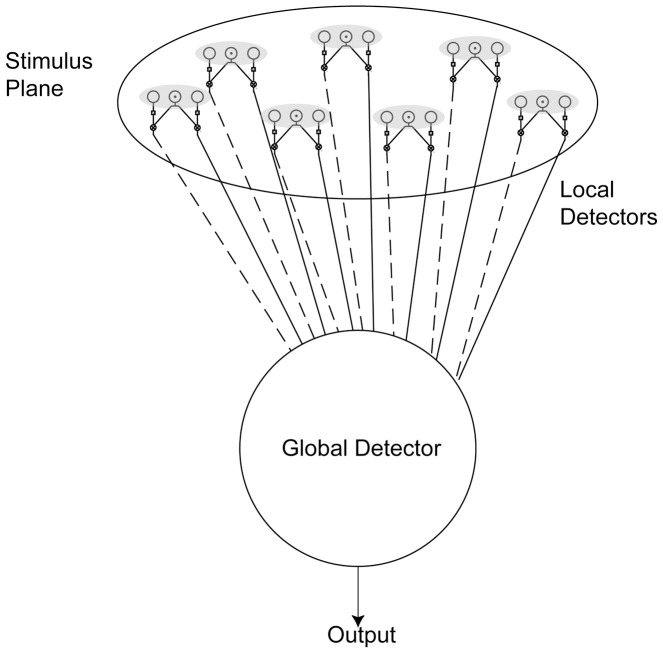
The integration of motion information. Schematic representing the collation of information from local detectors (based upon Reichardt detectors) into a global detector sensitive to a single axis of motion. Solid lines indicate excitatory connections and dashed lines, inhibitory connections. An excitatory summed response from the global detector in this case indicates rightwards motion.

Computer simulations were used to model the responses of the motion detecting system for the same stimuli as were used in the psychophysics. The model (Figure 1) simulated simplified local detectors (presumed representative of local motion selective cells found in area V1 (e.g. [53]), or area MT (e.g. [54], [55]) that tiled the stimulus plane, with their outputs feeding to a single global detector (presumed representative of global motion detecting cells found in area MT (e.g. [20], [21], [56]–[58])).

**Figure 2:**
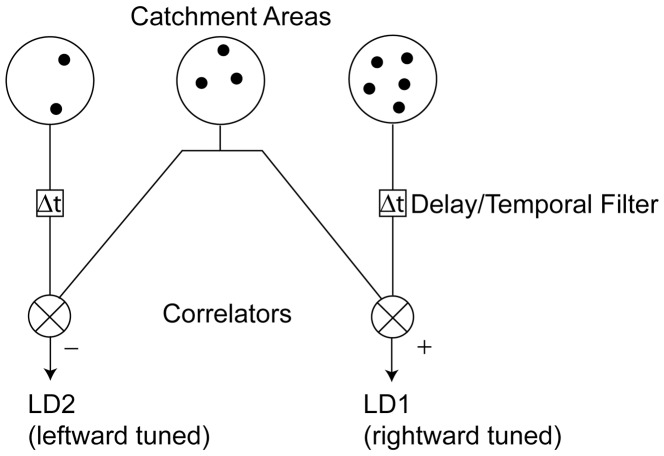
A modified Reichardt detector. This forms the basis of the local detectors in the module. Output from LD1 is excitatory and indicates rightwards motion. Output from LD2 is inhibitory and indicates leftwards motion. In this modified detector the central catchment area that samples in the neighbourhood of (x,y) in the first-field is shared between LD1 and LD2 (see text).

The local detectors are modified Reichardt detectors [59] that output the product of the activities in the two catchment areas (Figure 2), with the activity in each catchment area being proportional to the number of dots falling within the catchment area in the appropriate field. The model simulated local detectors sensitive to rightward and leftward motion. A detector for rightward (or leftward) motion would sample a location around the point (x, y) in the first field and around the point (x+δx, y) (or (x-δx, y)) in the second field, where δx is a positive quantity representing the dot displacement in the stimulus. The output of each local detector would be the product of the activities (i.e. the product of the numbers of dots) in the two catchment areas (see Figure 2), since this product represents the number of potential motion vectors linking the two catchment areas, as any of the dots from the first catchment area could have been displaced to any of the dot-locations in the second catchment area (see [1]). The modified Reichardt detector differs from the standard Reichardt detector in that the catchment areas for rightward and leftward local detectors are traditionally centred at ((x, y) and (x+δx, y)) and ((x+δx, y) and (x, y)) respectively. The justification for this modification is presented later in this section. An additional difference from the standard Reichardt detector is in the use of variable-size catchment areas, which can be thought of as rudimentary front-end spatial filters, yielding detectors closer to the elaborated Reichardt detectors proposed by van Santen and Sperling [60].

The catchment areas of the local detectors tiled the stimulus plane, using one of two tiling strategies. In one tiling strategy the number of local detectors was the same as the number of dots in the first frame of the RDK, and each first-field dot had one catchment area of a rightward-tuned local detector and one catchment area of a leftward-tuned local detector centred on it. The corresponding second field catchment areas were centred ±δx from the centre of the first-field dot. In the second tiling strategy, the first field catchment areas were randomly positioned over the first field, and the second field catchment areas were positioned ±δx from the centre of the first-field catchment area. The first strategy ensured that sampling (and performance) was optimal; every signal-dot pair was sampled at least once. The second strategy simulates the positioning of the human motion detectors, which cannot be reconfigured to match the positions of the stimulus dots. The number of local detectors was not a variable for the first tiling strategy (this number was the same as the number of first field dots), but could be varied in the second tiling strategy. However, in the main simulations of human performance using the second tiling strategy, the number of detectors was fixed at 1000 and was not permitted to vary freely when trying to match human and model performance.

There are two global motion detectors, tuned to rightward and leftward motion. The rightward (leftward) global detector sums the outputs of all the local detectors sensitive to rightward (leftward) motion. The model’s reported direction of motion for a given RDK stimulus corresponds to the direction of tuning (right/left) of the global detector with the larger of the two activities for that stimulus. The model’s response for a particular stimulus is considered to be correct if it matches the direction of coherent displacement of the dots between frames on that trial. The model’s proportion of correct responses for a particular stimulus-condition is determined from repeated presentations of instances of this stimulus.

The previously mentioned modification to the Reichardt detector used in the simulations was necessitated by the tiling strategy used in the simulations. When the tiling was optimal, the catchment area in the first field of the rightward detector was centred at a first frame dot. Using the modified detector ensured that even the leftward detector was centred at a first frame dot. If the traditional Reichardt detector had been used along with our optimal tiling strategy, this would have resulted in a rightward bias for motion in our local detectors, since our leftward detector would not be guaranteed to have at least one dot within the catchment area of the first field. In the interests of consistency, the modified Reichardt detector was used even when the tiling was random.

#### Assumptions of the model

The model described above makes several implicit assumptions, and these are listed here, along with some justification for these assumptions. The Appendix presents informal verification of some of the assumptions listed here.

##### 1. Local detectors that are optimally positioned outperform randomly positioned detectors

When the tiling of the stimulus plane follows the first of the two methods described in the previous section, every signal pair of dots is guaranteed to be sampled at least once. In addition, adding more local detectors to the simulations will not improve model performance, since the additional detectors will only be sampling information that has already been sampled at least once (i.e., no new information would be obtained by further re-sampling the same information). Simulation results presented in Appendix A confirm that the performance of the model cannot be improved beyond that obtained for optimally positioned local detectors simply by increasing the number of local detectors that are centred within the ESA and are randomly positioned with respect to the first field dots. Simulations with optimal tiling are useful for determining the performance of an ideal detector that uses all the information available in the stimulus, for the purposes of estimating absolute efficiency [1], [61]. In the current study the absolute efficiencies were found to be low and uninformative and therefore modelling of human performance in the main simulations was done with random tiling.

##### 2. Local detectors that have catchment area separations matched to the dot-displacement outperform detectors with catchment area separations that are mismatched to the dot-displacement

The separation of the catchment areas of the simulated local detectors matched the dot displacement in the stimulus. The implicit assumption here is that the motion detecting system contains banks of spatio-temporal filters tuned to different dot displacements [1], and the strongest response to the stimulus would be from the filters that are appropriately matched to the dot displacement in the stimulus. For the same reason, only horizontal motion detection was simulated, the assumption being that though detectors are available to detect motion in all directions, the detectors responding maximally to a horizontal motion stimulus would be those tuned for horizontal motion. See Appendix B for simulations that informally test this assumption. In these simulations we varied the mismatch between the separation of the catchment areas of the local detectors and the size of the dot displacement in the stimulus. The model performance was best when the separation matched the displacement size and systematically decreased as the separation was increased or decreased (Appendix B).

##### 3. Only local detectors which have their first field catchment areas centred within the ESA contribute to the global detectors

When collecting the psychophysical data, the observers always knew that the motion stimulus was confined to the central part of the total stimulus area. It is not clear that human observers could have confined their motion computations to the ESA, ignoring the flanking noise dots. However, having the first field’s catchment area centred within the ESA ensured that the local detectors picked up less of the noise and more of the motion information available in the stimulus.

##### 4. Radius of the catchment areas of the local detectors scales with size of the dot-displacement in the stimulus

In order to successfully capture the signal from a large dot-displacement, a motion detector would need a correspondingly large separation between its catchment areas. In our laboratory stimuli, the displacement of the dots is rigid, with all signal dots being displaced identically. However, in biological settings, deformations frequently accompany motion; all the spots on a sprinting leopard are not displaced the same distance over a brief time-interval. Scaling the radius of the detector catchment areas according to the size of the dot-displacement ensures a constant tolerance for non-rigid motion, regardless of the size of the average displacement in the stimulus. Thus the model assumes that the radius of the catchment areas (*R*) is given by the following equation:
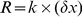
(1)where *δx* is the size of dot-displacement, and *k* is the constant of proportionality.

Appendix C describes how model performance is affected when *k* is increased. When *k* is small, the probability that any particular randomly-positioned local detector will be stimulated by a coherent pair of dots is reduced and more local detectors would be needed to adequately tile the stimulus plane. If an adequate number of local detectors are used, model performance is high for small values of *k*. As the value of *k* is increased, the catchment areas of the local detectors increase in a quadratic manner, as does the number of dots within each half of any local detector. This has the effect of rapidly increasing the number of potential motion vectors between the two catchment areas (even in the absence of an actual motion signal), and hence the correspondence noise increases. This results in a drop in performance with increase in *k* until for sufficiently large *k* motion detection is no longer reliable and Dmax is reached (Appendix C).

Equation 1 suggests that detectors tuned to larger displacements would have larger catchment areas. Since the temporal interval for optimal direction-discrimination performance (highest sensitivity) using 2-field RDK is 17–42 ms and is largely independent of displacement size [62], one interpretation of the above equation is that detectors tuned to larger velocities would have larger catchment areas and would hence be tuned to lower spatial frequencies. This is supported by psychophysical studies reporting shifts to lower spatial frequency mechanisms in the presence of image motion (e.g. [63], [64]) and by physiological studies of MT neurons that show preferred speeds for RDK stimuli that are highly correlated (*r* = −0.81) with preferred spatial frequencies of these neurons and less correlated with their preferred temporal frequencies (e.g Figures 10(E) and 10(F) in [65]).

When the first method of tiling (optimal tiling) was used for sampling the stimulus plane, *k* was the only free parameter in the model. When the stimulus plane was sampled by randomly positioned local detectors (random tiling), the additional parameter was the number of detectors used to tile the plane. However, across the different stimulus conditions simulated, this additional parameter was fixed at a pre-determined value of 1000 detectors (please see section titled *Preliminary Simulations*), so that, even in the case of random tiling, there was effectively only one free parameter when trying to model the psychophysical data. The objective of the simulations was to determine whether human performance for discriminating direction of motion in RDKs could be modelled across a wide range of stimulus parameters using effectively one free parameter.

##### 5. Minimalist approach to modelling could capture the essence of the computation involved

There are many factors that certainly contribute to human motion detection performance that the model does not attempt to represent. Some of these are:

- The profiles of the receptive fields of the motion detectors. All dots falling within the catchment areas in our simulations are weighted equally, i.e. the receptive field profile simulated is cylindrical. A more realistic simulation would involve Gaussian/Gabor receptive field profiles. However, it was felt that this would not affect the outcome of the simulations; the fundamental frequency of the cylindrical receptive field is the prime determinant of the spatial filtering properties of the model and the effects of varying the space constant of the Gaussian receptive field profile would be qualitatively similar to varying the radius of the catchment area. In addition, the saving in computation time on account of this simplification was enormous, particularly when the dot density was very high.
- The area of integration of the global detectors. The global detectors in our simulations integrate information over the entire stimulus area, whereas the area over which integration of motion information is efficient has been psychophysically determined to have a radius of approximately 2 degrees [1]. This radius matches well with the receptive field sizes measured for global motion detectors in MT/V5 [66]. However, the variation in receptive field sizes reported for MT neurons in different studies is large (e.g. [9], [22], [53], [67]–[69]).

The above factors (among others) have an influence on human motion detection performance, but including these factors in the model would introduce many more free parameters in the simulations. We anticipated that the absence of a representation of these in our minimalist model could be compensated for by appropriate adjustments to *k*. Therefore, the radius of the catchment area (*R*) obtained from the simulations should be seen as the *effective* radius, which simulates the consequences of spatially scaling the receptive fields of motion detectors, along with other factors that are not explicitly represented in the model.

#### Comparison with previous models/approaches

Several similarities and differences exist between the model simulated here and previous approaches to simulating motion in RDKs:

##### 1. Low-pass filtering

Previous studies have suggested low-pass filtering of the individual fields before feature matching across the filtered images [40], [70]. The summing of the dots within each catchment area is equivalent to low-pass filtering the image; the larger the radius of the catchment radius, the lower the cut-off frequency.

##### 2. Feature matching

The feature matching stage of some of the earlier models (e.g. [40], [70]) is entirely eliminated in the current simulations. This does away with the need for an algorithm for feature matching in the filtered images. In this respect, the simulations here are similar to those of [43].

##### 3. Banks of spatio-temporal filters

The human motion detecting system is presumed to consist of banks of spatio-temporal filters [1]. Though only one set of filters are simulated, the assumption made is that there exist banks of filters and the one simulated, and hence used in the decision making process, is the one that is expected to be most sensitive to the motion information available in the stimulus.

##### 4. Information limit/correspondence noise limit

When the tiling of the stimulus plane is optimal, the performance of the model is at its informational limit for a particular catchment area radius (*R*). This is tested systematically in Appendix A.

#### Preliminary simulations

The model’s proportion of correct responses for a particular set of stimulus parameters was determined by repeatedly presenting the model with instances of stimuli having these fixed parameters and determining the proportion of trials for which the model correctly identified the direction of motion. Simulations indicated that 1000 repetitions (or trials) were adequate to ensure repeatable model performance (see Appendix A for simulation results). These simulation results also indicated that model performance with optimal tiling of the stimulus plane by the local detectors exceeded the model performance with randomly positioned tiling of the local detectors, as predicted above. In the simulation results presented in the rest of this paper the local detectors randomly sampled the stimulus plane. This was because the model performance with optimum tiling was superior to human performance, particularly when the coherence level was low (data not presented here, see Shafiullah, 2008). Furthermore the simulations showed that model performance was close to its asymptotic level when 1000 local detectors were used, and this number of local detectors was used in all of the stimulus conditions of the main simulations, so that the number of detectors was not a free variable when matching psychophysical performance to model performance.

## Results

**Figure 3:**
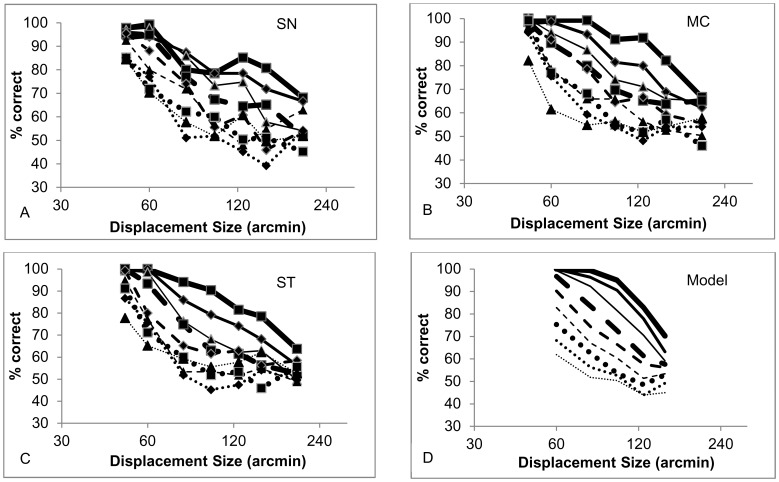
Effect of dot displacement size on human and model performance. Direction discrimination performance with an RDK and right vs left motion for three observers (panels A–C) and the correspondence noise model (panel D) as a function of dot displacement size, with dot density as a parameter. Squares and thick solid line: 0.13 dots/deg^2^, Diamonds and medium solid line: 0.27 dots/deg^2^, Triangles and thin solid line: 0.53 dots/deg^2^, Squares and thick dashed line: 1.07 dots/deg^2^, Diamonds and medium dashed line: 2.13 dots/deg^2^, Triangles and thin dashed line: 4.27 dots/deg^2^, Squares and thick dotted line: 8.53 dots/deg^2^, Diamonds and medium dotted line: 17.07 dots/deg^2^, Triangles and thin dotted line: 26.67 dots/deg^2^.

The results of the observer’s performance for the direction discrimination task in the RDK across a wide range of conditions, but when the coherence level is 100%, are plotted in Figure 3A–C. These show that, unless the conditions were such that the performance was consistently nearly perfect (e.g. for smaller displacements between 1 arcmin and 40 arcmin), in which case the data is not shown, the probability of correctly identifying the direction of motion for any given displacement diminishes as the dot density increases across more than two orders of magnitude.

These data were then used to estimate Dmax for each observer at each dot density. A graph of performance as a function of displacement was produced for each dot density. A cumulative Gaussian function, scaled to fit between 50% and 100% correct, was fitted to this data by varying the standard deviation (slope) and mean (75% correct threshold) of the Gaussian. The displacement corresponding to 75% correct performance estimated from the fitted Gaussian was used to represent Dmax.

**Figure 4:**
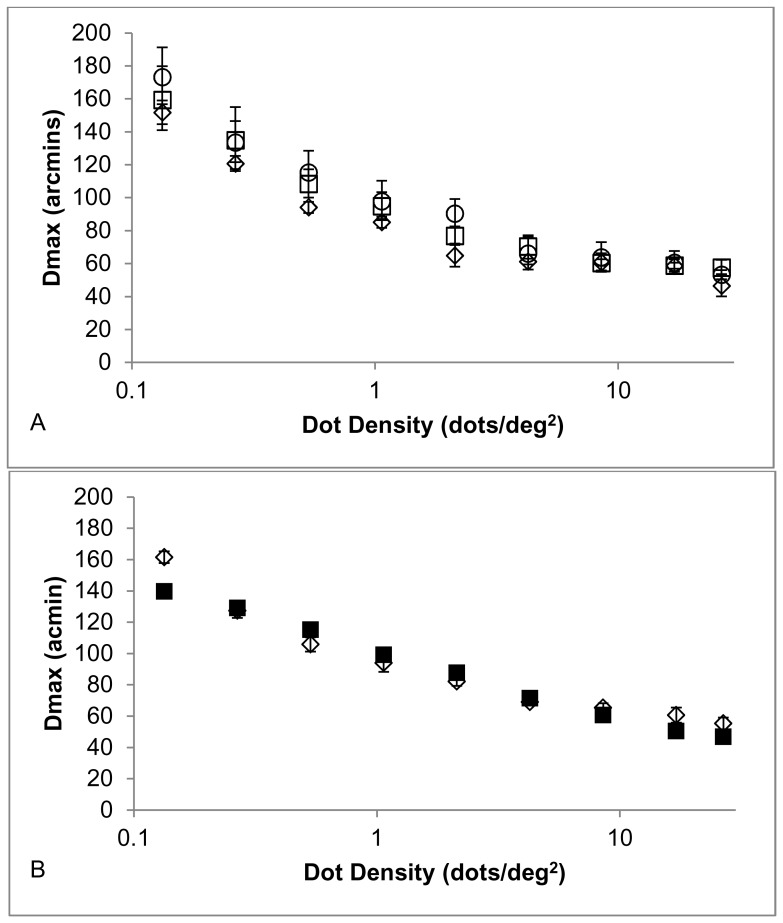
Effect of dot density on human and model Dmax. A. The value for Dmax derived from the raw direction discrimination performance data. Dmax is plotted as a function of dot density for each of the three observers. Open circle MC; open square SN; open diamond ST. Error bars show the 95% confidence interval for Dmax derived from the fitting procedure. B. Open diamonds show the Dmax values derived from the average performance of the three observers with the filled squares indicating the values of Dmax derived from the model results of direction discrimination performance as a function of displacement size. The error bars are as for the top panel. When error bars are not present, errors are smaller than the symbol size.

The estimated values of Dmax for each observer were then plotted as a function of the dot density in Figure 4A. This showed that Dmax diminished as a function of dot density in a predictable way, well represented by a power function with a negative exponent.

**Figure 5:**
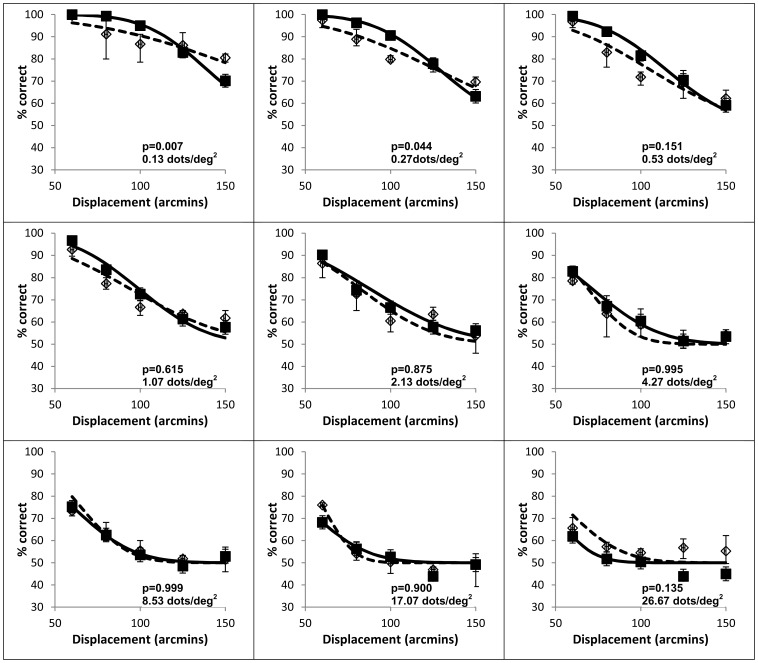
Comparison of human and model performance for different dot densities. Psychophysical (open symbols) and model (closed symbols) direction discrimination performances are plotted as a function of displacement size. Panels, reading from left to right, then top to bottom, display data from dot densities of 0.13, 0.27, 0.53, 1.07, 2.13, 4.27, 8.53, 17.07 and 26.67 dots/deg^2^. Model *k* = 1.88; Coherence level = 100%; 1000 random local detectors were incorporated in the model. Psychophysical performances were the average of three human observers and the error bars show the range. Model performances were a result of 1000 trials at each data point and the error bars represent the 95% confidence interval. A fitted cumulative normal function is shown by the solid lines (model data) and dashed lines (human data). The p-value of the χ^2^ statistic was measured to determine the strength of association between the psychophysical and model data and is displayed for each density.

At the level of the raw data, i.e. the direction discrimination performance for a known dot displacement and dot density, it was possible to use the model to generate equivalent data once the model had been constrained by a single parameter, the value of *k*. From this it was possible to produce the model fits that are shown in Figures 3D, 4B and 5 at different levels of the data analysis.

### A. The Effect of Dot Density on Performance

Figure 3, panels A–C, shows the direction discrimination performance from each of the three observers as a function of displacement size, with dot density as a parameter. Figure 3 panel D shows the fit of the model, when *k* was set to its optimum value of 1.88 for fitting across all the data for the range of testing conditions and averaging the performance across the observers (see section below titled “Simulations of motion perception performance” for details regarding the optimisation of *k*). This was believed to be justified as the individual differences in performance were small compared to the effects of the parameters under investigation, namely displacement size and dot density. To avoid clutter, 95% confidence intervals for the data points are not shown but range from ±8% at performance levels near the chance level of 50% to ±0% at 100% performance.

Each observer follows a similar pattern, with performance diminishing with increasing dot density and with increasing displacement size. The psychophysical data curves appear to move to the left and steepen slightly in slope as dot density is increased. The model’s performance show the same qualitative effects of displacement size and dot density and match the slopes of the functions reasonably accurately, considering the level of uncertainty in each datum point and that the model has been fitted to an average of the observers (see Figure 5 for data showing average performance of the three observers) rather than each individual. The position of each model fit curve along the abscissa does not, however, match the psychophysical data for the individual observers so well and it is evident that the model requires further tuning to better match individual performances. The scale of the effect of the dot density and displacement size on performance was confirmed statistically using a repeated measures two-way ANOVA on the psychophysical data from the three observers. This showed significant effects of dot density (F_8,126_ = 92.4, p<10^−48^) and displacement size (F_6,126_ = 245.8, p<10^−66^) as well as a significant interaction (F_48,126_ = 3.44, p<10^−7^). From the significant interaction it is evident that the performance curves are not simply shifted versions of one another and that a successful model must describe both the change in abscissa position and the change in the slope of the function as dot density is varied.

### B. Estimating Dmax from Direction Discrimination Performance

Both the modelled data and the psychophysical data were fitted with cumulative Gaussian functions as explained earlier, and the displacement that represented the 75% correct point on these fits was used to define the value of Dmax for the given dot density and observer (or model). This allowed us to investigate the effect of dot density on Dmax for both the psychophysical data and for the modelled data.

### C. Effect of Dot Density on Dmax

Figure 4 shows how Dmax depends upon the dot density. In the upper panel, showing the psychophysical results from the three observers, the most apparent feature is the consistent reduction in Dmax as dot density increases followed by a more gradual reduction in Dmax as dot density increases further. This feature would be even more apparent if dot density were plotted on a linear scale. The individual differences in Dmax are also apparent, especially at the lower dot densities where Dmax ranges between approximately 150 and 175 arcmin.

The lower panel of figure 4 compares the averaged human direction discrimination performance to the model direction discrimination performance, by plotting experimental and model values of Dmax as a function of dot density. When this is done, the model closely corresponds with the human data over a three-fold change in Dmax and a 200-fold change in dot density. Only at a dot density of 0.13 dots/deg^2^ do the two data sets differ noticeably.

### D. Simulations of Motion Perception Performance

Figures 3D and 4B show model performance once the optimal value of *k* ( = 1.88) had been determined. The procedure for determining this optimal value of *k* is described below. The process to find the optimum value of *k* for the model began by visually selecting the value of *k* that gave the best match between the direction discrimination performance of the model and the average human observer across the range of dot densities and displacement sizes for which data was available. In this part of the modelling, the different values of *k* were sampled very coarsely. This visually determined value of *k* formed a preliminary estimate of the scaling factor. Starting from this point, a range of five separate *k* values in steps of ±25% (0.1 log units, or 1dB) were chosen and the model data generated from them at displacement sizes of 60, 80, 100, 125 and 150 pixels and dot densities of 20, 80, 320, 1280 and 4000 dots per ESA. The ESA was sampled with 1000 randomly position local motion detectors and the direction discrimination performance across 1000 trials of each combination determined. A sum of residual error (expressed with a χ^2^ statistic) between the model and the average human data was calculated at each value of *k* and these residual errors were plotted as a function of *k*. This produced a U-shaped function that was fitted with a quadratic equation. The *k* value at which this function reached a minimum was chosen as the best fitting value of *k*.

Whilst the results from the modelling in aligning to the raw and derived data in the figures presented so far have been supportive of the model as an explanation for human visual motion direction discrimination performance, it would be interesting to see more explicitly the performance of the model under the range of conditions under test, and to find a statistical metric to evaluate its effectiveness at explaining the human data. To that end, figure 5 is presented below.

When interpreting this figure it is important to note that only one parameter in the model, *k*, was available to modify to determine the best fit across all nine functions to the data and that an average of the direction discrimination performance across the three observers was used. The dashed and solid lines, representing the best fitting cumulative Gaussian function to the data from the average observer and from the model respectively, lie close to the data points in all the functions except for at the highest dot density of 26.67 dot/deg^2^ where human performance asymptoted above 50%.

The model fit was found to be an adequate fit to the human performance data in 7 of the 9 functions (p-values indicated in the individual panels of Figure 5). The model failed to satisfactorily explain the human data at dot densities of 0.13 and 0.27dots/deg^2^. In each case the model overestimated the human performance at lower displacement sizes and fell as a function of displacement size with a steeper slope. With these very sparse patterns and large displacements the possibility of opportunistic feature tracking arises, and this could preserve direction discrimination performance beyond that predicted by considering only the correspondence noise.

**Figure 6:**
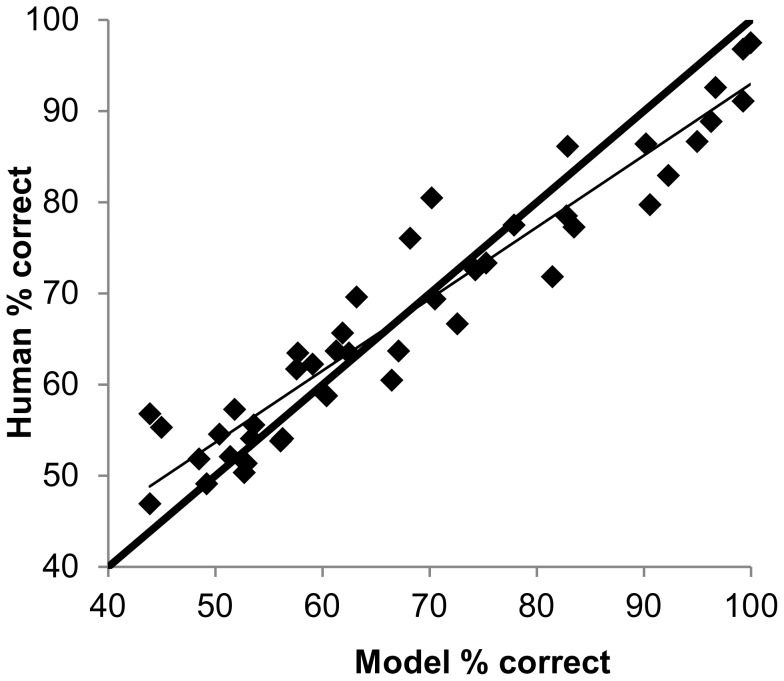
Comparison of human and model performance across all conditions. Psychophysical vs model direction discrimination performance pooling data from nine dot density levels and five displacement levels. The thick solid line is the line of equality whilst the thin solid line is the line of best fit, where human performance = 0.79 × model performance +14% (r^2^ = 0.91).

Figure 6 is a summative figure plotting the human direction discrimination performance against the model direction discrimination performance, using data from all 9 dots density levels and the 5 displacement levels that were modelled. All points lie close to the line of equality, shown by the solid thick line, and the data are well represented by a linear fit where performance = 0.79 × model performance +14% (r^2^ = 0.91). There is a marginal trend for the model performance to exceed that of the human observers when performance levels are high and for human performance to exceed that of the model when performance is close to chance. A closer inspection of Figure 5 indicates that the largest differences between human and model performance are seen for lower dot densities (first three panels) and smaller displacements (<100 arcmins). These data would be mapped to the data points that fall furthest from the line of equality towards the upper-right quadrant of Figure 6, i.e. data corresponding to high human and model performance. A potential explanation for the mismatch in performances at lower dot densities and smaller displacements is that the global detector in the model, which integrates information over the entire stimulus, might be relatively more efficient than the human global motion detectors that integrate efficiently over regions of radius of approximately 2 deg [1]. The model’s relative advantage in efficiency is presumably lost when the displacements become large (as do the local detectors) and/or the dot density becomes large as the model integrates increasingly noisy motion information; the human observers integrate relatively less noisy information due to the smaller region of integration of the global motion detectors. In summary, the model performance closely matches that of human observers and any difference in the two is most likely to be a consequence of the model having a single global detector for each of the two directions of motion.

## General Discussion

Barlow and Tripathy [1] systematically investigated the influence of correspondence noise on threshold coherence in kinematograms. The current study extends our understanding of the influence that correspondence noise has on Dmax in kinematogram stimuli which have all of the dots moved coherently. Psychophysical performance measures were obtained across a wide range of dot densities and displacement sizes, from which Dmax was estimated over a wide range of dot densities. A minimalistic model was proposed to compute performance based on the correspondence noise in each stimulus. The model had two parameters, the number of local detectors at the front end of the motion detecting system that have receptive fields overlapping the stimulus and the scaling factor *k* which determines the range of displacements to which a particular detector responds. The number of detectors used in the simulation was selected, independently of any attempts at matching human performance data, by choosing a number giving arbitrarily close performance to the asymptotic level (see *Preliminary Simulations*). Only *k* was adjusted in order to find a *single* value that would optimise matching between the model and the human data across the entire stimulus space investigated. This minimalistic approach successfully simulated motion discrimination performance across a wide range of stimulus parameters (Figures 5 and 6).

The following sections discuss: the relation of this work to previous psychophysical work on motion detection, the links between the model proposed and earlier simulations of Dmax, neurophysiological implications of the current work, and implications for statistical efficiency.

### A. Relation to Previous Psychophysical Work

Several studies have investigated the effect of dot density on Dmax (e.g. [5], [42], [43], [71], [72]). Most of these studies found that Dmax increased systematically with decreasing dot densities, particularly when the dot densities were very low. The exception was the study by Baker and Braddick [5], which found no effect of dot density on Dmax.

**Figure 7:**
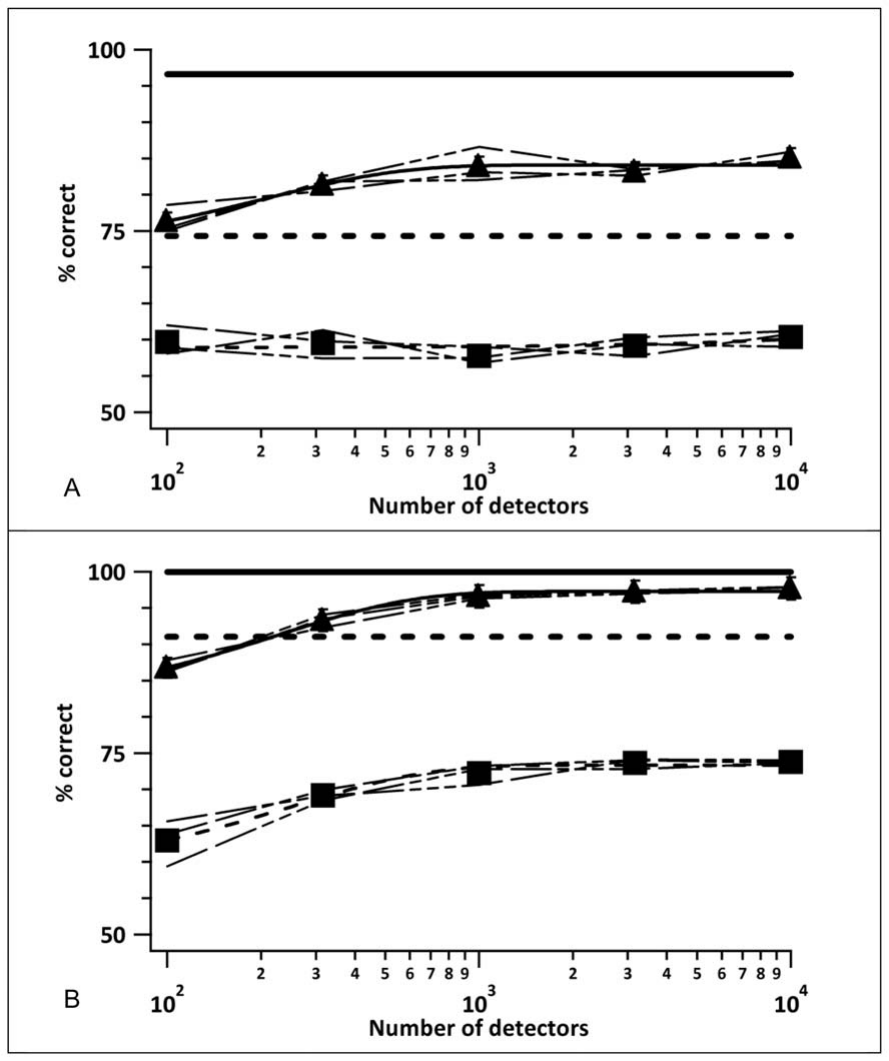
Effect of number of detectors on model performance. Modelled direction discrimination performance with an RDK and right vs left motion for two displacement sizes (220 arcmin in panel A; 90 arcmin in panel B) and two dot densities (500 dots – filled triangles and horizontal solid line; 4000 dots – filled squares and horizontal dashed line) as a function of the number of local detectors used to tile the effective stimulus area. Horizontal lines show the performance when the local detectors optimally tiled the stimulus plane. Thin dashed lines represent three repetitions of 1000 trials each for each value of dot number and show repeatable model performance. The thicker curves and the filled symbols represent the means of the three repetitions. Error bars are ± one standard error.

The most systematic study of the effect of dot density on Dmax was by Eagle and Rogers [42]. They measured Dmax for patch sizes varying between 2.6 and 645.2 deg^2^ and for dot coverage (i.e. the proportion of the stimulus area occupied by the dots – sometimes referred to as “dot density” in the literature) varying between 0.025 and 50%. They found that dot coverage/density had little effect on Dmax for their smallest patch sizes, but had a substantial effect on Dmax when patch size was increased (also see [43]). Eagle and Rogers [42] and Sato [43], both, attributed the absence of an effect of dot density in the Baker and Braddick [5] study to the small patch size (1.53×0.77 deg) used in that study.

The stimulus in the current study had an ESA of 150 deg^2^ and dot coverage ranging from 0.06% (20 dots) to 23.7% (4000 dots). The stimulus that was the closest match from the Eagle and Rogers [42] study was one with a patch height of 12.7 deg (area of 161.3 deg^2^) in their Figure 3. For this patch size and for our range of dot coverage, the average Dmax for the three observers in the Eagle and Rogers [42] study had a range ≈135–50 arcmin. The corresponding range of Dmax in our study was 161–55 arcmin. In addition, when Dmax and dot density are plotted on log-log axes, Eagle and Rogers reported ([42], p. 2096) a slope of −0.2, which is close to the slope of −0.22 seen when the lower panel of our Figure 4 is replotted on log-log axes. In spite of differences in stimulus conditions Dmax measures from the two studies are in reasonable qualitative and quantitative agreement.

**Figure 8:**
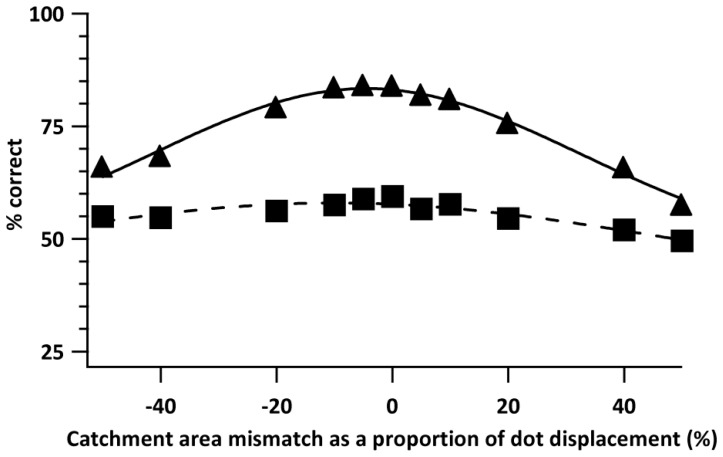
Effect of stimulus-detector mismatch. Modelled direction discrimination performance with an RDK and right vs left motion for 220 arcmin displacement size and two dot densities (500 dots – filled triangles and solid line; 4000 dots – filled squares and dashed line) as a function of the mismatch between the local detector’s catchment area separations and the stimulus’ dot displacement. Lines are fits of Gaussian functions to the data.

The simulations here suggest that the detection of large displacements requires large detectors; large detectors have large catchment areas; it is the noise from these large catchment areas that limits performance.

### B. Relation to Previous Simulations of Dmax

Several studies have proposed a low-pass filtering of the RDK stimulus before motion detection takes place [40], [42], [43], [70]. In our modelling, the size of the catchment area of the local detectors determines their spatial frequency tuning; the larger the catchment area, the less sensitive it is to local variations in dot density or to spatial detail. Counting the number of dots in each catchment area is the equivalent of spatial filtering the stimulus; taking the product of the number of dots in the two catchment areas is equivalent to evaluating the motion in the local detector by counting the number of potential motion vectors it sees. Thus our model, which counts the number of dots in the two catchment areas and multiples them, is analogous to the detection of motion in low-pass filtered RDKs of earlier studies.

**Figure 9:**
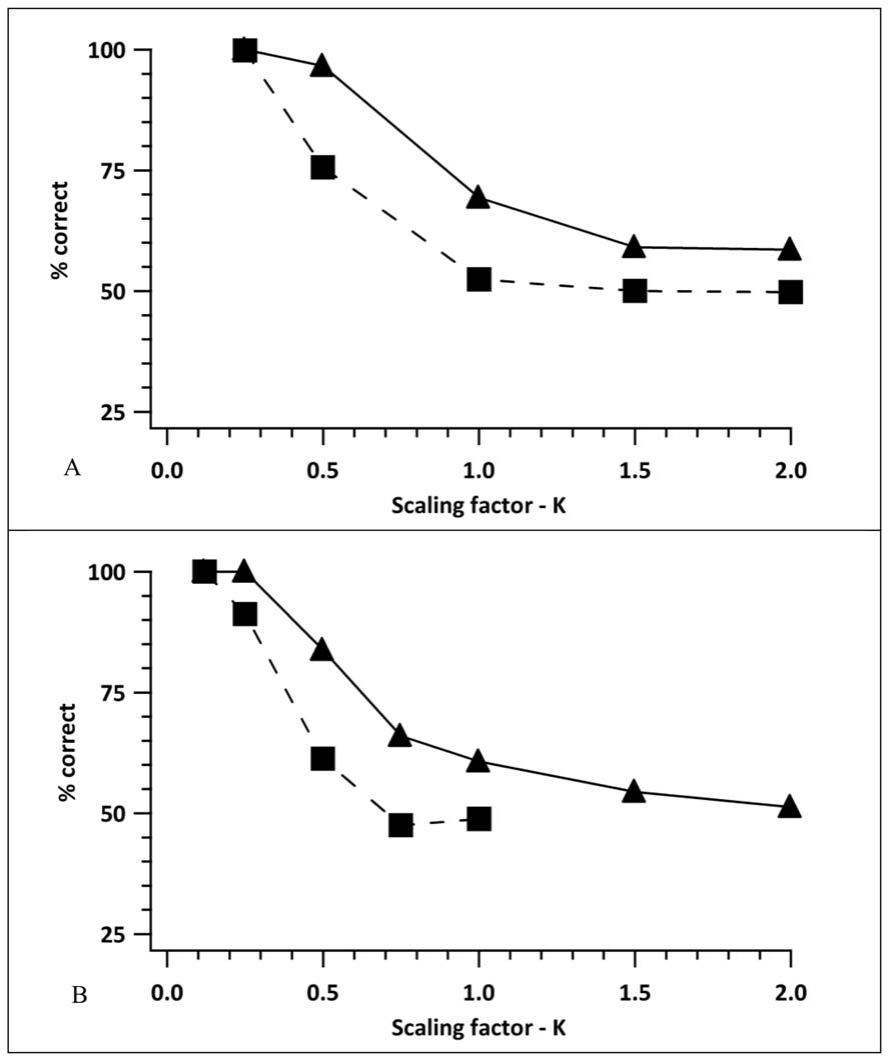
Influence of scaling factor (k) on model performance. Results for the model performance for a large range of *k* tested under two different local-detector-tiling conditions: Panel A - optimally positioned local detectors; Panel B - randomly positioned local detectors with the number of local detectors set to 1000. Two dot-densities were used in each tiling condition (500 dots – filled triangles and solid line; 4000 dots – filled squares and dashed line).

Though there are several similarities between our simulations of Dmax and earlier ones, there are important differences. The most elaborate of the previous simulations are presented in Eagle and Rogers [40], [42], and a comparison with these earlier simulations will help to illustrate the novelty of our approach. While the current approach represents significant improvements in the modelling of Dmax, it must be recognised that the papers of Eagle and Rogers [40], [42] provided very fundamental insights into disambiguating the “phase hypothesis” from the “informational hypothesis”. A comparison of the two approaches follows.

#### 

##### 1. Model Complexity

In these earlier studies the stimulus was pre-filtered with oriented Gabors and peaks were identified in the filtered images; Dmax was proposed to be proportional to the mean 2-D spacing of the peaks. The complexity of the earlier model means that there were a number of free parameters available for fine-tuning the response: the Gabors are simulated as a difference of two Gaussians, yielding two free variables for the centre and surround space constants (σ_c_ and σ_s_) and one free variable (β) for the standard deviation of orientation tuning; the constant of proportionality *k* that links Dmax to the mean 2-D spacing of the peaks is an additional free variable. In contrast, there were only 2 variables in our simulations: the number of local detectors and the scaling factor *k*, and, after preliminary explorations of the parameter space the first of these was fixed at 1000 detectors, while the second was a free variable that was used for simulating the *entire* data set. This does not imply that spatial filtering does not occur in the motion detectors of the human visual system. The goal of the current simulations is to capture the essence of the computations underlying Dmax, in particular, the scaling of the detector-size with increase in the size of dot displacement, and the resulting increase in correspondence noise and loss of performance. Adding spatial frequency filters to the front end of the local detectors would have made the simulations more realistic and improved the match between human and model performance. However, our minimal model highlights the principles that the simulation intends to capture.

##### 2. Match of psychophysical and simulation results

Eagle and Rogers [42] found their simulation results did not match their psychophysical data qualitatively (compare their Figures 3 and 6), and invoked contrast-effects at low dot densities in order to explain the difference between data and simulations. In spite of our minimalistic approach to modelling, our simulations were effective in modelling the psychophysical data (our Figure 6). The simulations had short-comings and these are evident in Figure 6, with the model systematically underestimating the human performance when performance is close to chance (i.e. when displacements are significantly greater than Dmax) and overestimating human performance when performance is close to saturation (i.e. when displacements are smaller than Dmax). However, our simple model accounted for 91% of the variance in human performance across the psychophysical parameter space explored.

##### 3. Performance for displacements different from Dmax

The earlier studies make a point-value prediction of Dmax, (i.e. given a stimulus with a particular dot-density, the model proposed extracts the peaks in the filtered stimulus, and, from their spacing, estimates Dmax), however, the model does not predict performance of the human observer if the dot displacement in the stimulus was 0.5×Dmax or 1.5×Dmax. In contrast, the model proposed here estimates Dmax by predicting the psychometric function as a function of dot-displacement, as seen in Figure 5, and can predict human performance for dot-displacements of a fraction, or a multiple, of Dmax.

##### 4. Filter characteristics

The earlier studies utilised a *single* set of filter characteristics to model Dmax; the size of the filter does not change when the size of dot-displacement changes. The current study assumes that there are *banks* of filters tuned to different dot-displacements and the filters that are most responsive to a particular dot-displacement are the ones tuned to that displacement. Even though for any stimulus, the model response is based on the output of the filter most sensitive to the coherent displacement in the stimulus, the assumption is that there are a host of filters in the visual system that are tuned to other displacements that are not represented in our simulations because we incorporate a *winner-take-all* strategy. In addition, the size of the filter is scaled with the size of the displacement.

##### 5. False matches

In the earlier model the false matches from intervening dots between the two positions occupied by any signal dot reduce Dmax. In contrast, in the current model, the critical variable is not how many false matches lie between the two positions occupied by a signal dot, but how many mismatches lie close to the two positions across which co-relations are sought. Eagle and Rogers [42] had difficulty explaining why their constant of proportionality linking Dmax to the mean element spacing was much larger than the maximum of 0.5 that would be predicted for nearest-neighbour matching. In the current model the nearest-neighbour concept has less significance compared to the earlier model, and when *k* <0.5, intervening dots that lie outside of the catchment areas would make no contribution to the noise. However, this distinction between the two models is obscured when *k* >0.5 (as in the current simulations), since dots lying between the two positions of a signal dot also lie within the bi-local catchment areas of the local detector centred on those two positions.

One of the short-comings of the model proposed in this study is that it is not very realistic. There is only a single global detector for each direction of motion and this is exactly matched to the stimulus area. Having many global detectors and a mechanism for combining information across these global detectors would make the simulations more realistic, but would substantially increase the number of free parameters in the model. While the addition of free parameters would permit modelling the data more accurately, it is likely that the complexity of the model would have masked the simplicity of the computation. In our simulations all of these additional potential complexities have been incorporated into a single parameter *k,* and this simplified model gives a very good account of the data.

### C. Implications for the Neurophysiology of Motion Detection

Barlow and Tripathy [1] proposed a vast array of filters in cortical area MT, with each filter responding to different combinations of parameters, such as size, direction of motion, etc. The current study attempted to model Dmax assuming an array of detectors tuned to different displacements. The success in modelling Dmax with the simple model used here adds support for the filter arrays proposed above. The large value of *k* ( = 1.88) used in the simulations indicates that the detectors used were much larger than the size of the displacement. Our detectors were approximately 4.76 (L) and 3.76 (W) times the size of the displacement in the stimulus. The large value of *k* is also consistent with broad orientation tuning of the local detectors and the global detectors that sum the outputs of these local detectors. The large detectors and the broad directional tuning are both predicted by the coarse tuning of motion detectors reported in Barlow and Tripathy [1].

The simulations suggest that detectors that are tuned to large displacements have large catchment areas, i.e. are tuned to low spatial frequencies. This might explain why experiments that have attempted to understand Dmax using spatially filtered stimuli, have frequently yielded conflicting results. Most of the experiments with filtered RDKs used fixed cut-off/centre frequencies for low−/high−/band-pass filtering the stimulus (e.g. [52]). But the simulations suggest that the spatial frequencies that are critical to the stimulus change with the size of the displacement in the stimulus. Therefore to fully understand the spatial frequencies that are critical for direction discrimination, the design of experiments that use spatially filtered RDKs should take into account the size of displacement of the dots in the stimulus when selecting the filter characteristics.

### D. Implications for Statistical Efficiency

Barlow and Tripathy [1] reported efficiencies of about 30% for detecting coherent motion under coarsely quantised stimulus conditions. A variety of potential factors were listed as to why the efficiency, even for the most coarsely quantised stimuli tested, was well below 100%. The current study identifies an additional source of efficiency loss to those identified in the previous study. Appendix A shows that the model performed much better when the local detectors were positioned optimally, compared to when the detectors were randomly positioned. In the ideal observer calculations in Barlow and Tripathy [1], it is presumed that motion detectors are available at the appropriate positions to make optimal use of the information presented in the stimulus. However, the positioning of detectors in the human observer’s motion system is unlikely to be optimally matched to that of the stimulus. This could result in significant losses in efficiency in the human motion detection system.

## Appendix

This Appendix summarises some of the preliminary simulations conducted in order to understand the performance of the model. These simulations explored the consequences for model performance when varying variables such as the number of trials to be simulated, the number of detectors to be used in the simulations, the type of tiling, the mis-match between detectors and stimuli, etc. Since these were exploratory simulations that were conducted prior to actually fitting model performance to psychophysical performance, some of the parameters used in the simulations below do not match the parameters used in the simulations described in the Results section. However, the simulation results below illustrate the motivation behind some of the modelling choices made and provided us valuable insight when performing the simulations described in the section on Results.

### A. Estimating the Appropriate Number of Trials and Local Motion Detectors for Each Stimulus Condition

Simulations were carried out to estimate the number of repetitions (or trials) necessary in order to ensure repeatable model performance. The stimuli input to the model had 500 or 4000 dots presented within the ESA, with dot displacements being 220 arcmin or 90 arcmin across the two fields. The simulations used the following model parameters: *k* was 0.5 (220 arcmin displacement) or 1.0 (90 arcmin displacement); the number of repetitions was set to 1000; the number of local detectors used in the simulation was 100, 300, 1000, 3000, or 10000 when tiling was random, and was the same as the number of stimulus dots (500 or 4000) when the tiling was optimal. For each combination of number of stimulus dots, stimulus dot displacement, and number of local detectors in the model, three repetitions of 1000 trials each were conducted and the proportion of correct responses over each set of 1000 trials was determined.

Figure 7, panels A and B show the simulation results for the 220 and 90 arcmin displacements respectively, with the number of local detectors plotted on the abscissa and the model’s proportion of correct responses along the ordinate. The three dotted curves along the filled triangles represent three repetitions of the simulations for the 500-dots stimulus and the solid curve represents the mean of the three individual curves. The three dashed curves along the filled squares and the accompanying short-dashed curve represent the equivalent simulation results for the 4000-dots stimulus. The error bars, all smaller than the symbol height, represent ±1 SE. The horizontal solid and short-dashed lines are the simulation results, when the tiling was optimal, for the 500- and 4000-dots stimuli, respectively. Note that the numbers of detectors in these simulations were 500 and 4000 respectively, but they are shown as a line to facilitate comparison with the asymptotic performance when the local detectors randomly tile the stimulus plane. The overlap of the three repetitions of the simulations, and the SEs indicate that 1000 trials were adequate to obtain repeatable results and subsequent simulations used 1000 trials for each stimulus condition. In addition, subsequent simulations used 1000 local detectors per stimulus as this gives near asymptotic performance levels in each case.

It is apparent from Figure 7 that optimally positioned local detectors outperform randomly positioned local detectors, even when the number of randomly positioned detectors far exceeds the number of dots in the stimulus. This assumption is more thoroughly tested in [47].

### B. Local Detectors that have Catchment Area Separations Matched to the Dot-displacement Outperform Detectors with Catchment Area Separations that are Mismatched to the Dot-displacement

In these simulations a mismatch was deliberately introduced between the catchment area separations in the model and the size of the dot displacement in the stimulus. We anticipated that performance of the model would drop as the mismatch was increased.

The stimulus consisted of 500- or 4000-dots RDKs with a dot displacement of 220 arcmin. The simulations used 1000 randomly-positioned local detectors, with *k* = 0.5. The mismatch of the local detector’s catchment area separations to the dot displacement was ±5%, ±10%, ±20%, ±40%, or ±50% of the dot displacement.

Figure 8 plots the percentage of correct responses as a function of the mismatch of the catchment area separation to the size of dot displacement. For the two dot densities, the Gaussian fits to the data clearly indicate that the peak performance occurs at 0% mismatch, i.e. when the separation of the catchment areas matches the dot displacement.

### C. Effect of k on Model Performance

In the simulations above *k* has been fixed at 0.5. Whilst this appears to be a reasonable starting point, it is important to get an idea of how the value of *k* influences the model performance over the range of values at which we might be modelling. To this end, for a known set of model stimulus conditions, i.e. dot densities of 500 dots and 4000 dots within the effective stimulus area, and a displacement size of 220 pixels, we used 1000 trials to estimate the model performance when the local detectors were positioned optimally (in this case the number of local detectors matches the number of dots) and when 1000 local detectors were positioned randomly. The results are shown in panels A and B respectively in Figure 9.

As seen previously, performance with the lower dot density is consistently better, and it is of note that the shape of the various curves showing how performance falls as *k* is increased is very similar across conditions. Essentially, it should be possible to match different model designs (optimal detector placing or random detector placing) simply by altering the value of *k* that is used.

As expected, increasing the value of *k* increases the correspondence noise affecting the local motion detector and reduces performance. It appears from panel B, where the local detectors are randomly placed and mimic what we would expect to be happening in the human visual system, that values of *k* in excess of 1 would not provide motion direction discrimination performance above chance when the dot density is high. This limitation reflects the large displacement size that is modelled here as human observers were incapable of determining the direction of stimulus motion in cases where the displacement size was beyond 150 arcmin. By lowering the displacement size, the range of values of *k* that may be used in fitting human data will be extended at these high dot densities.
